# The World Rugby and International Rugby Players Contact Load Guidelines: From conception to implementation and the future

**DOI:** 10.17159/2078-516X/2023/v35i1a16376

**Published:** 2023-12-20

**Authors:** LT Starling, R Tucker, K Quarrie, J Schmidt, O Hassanein, C Smith, S Flahive, C Morris, S Lancaster, S Mellalieu, O Curran, N Gill, W Clarke, P Davies, M Harrington, E Falvey

**Affiliations:** 1 World Rugby House, Pembroke Street Lower, Dublin, Ireland; 2 Department for Health, University of Bath, Bath, UK; 3 International Rugby Players, Clonskeagh, Dublin, Ireland; 4 C J Morris Consulting Ltd, Cheshire, UK; 5 New Zealand Rugby, Wellington, New Zealand; 6 Leinster Rugby, Belfield, Dublin 4, Ireland; 7 Centre for Health, Activity and Wellbeing Research (CAWR), Cardiff School of Sport and Health Sciences, Cardiff Metropolitan University, Cardiff, UK; 8 Institute of Sport and Exercise Medicine (ISEM), Department of Exercise, University of Stellenbosch, South Africa; 9 Irish Rugby Football Union, High Performance Centre, National Sports Campus, Dublin 15, Ireland; 10 University of Waikato, Tauranga, New Zealand; 11 College of Medicine & Health, University College Cork, Cork, Ireland

**Keywords:** rugby union, contact training, load

## Abstract

Managing training load in rugby union is crucial for optimising performance and injury prevention. Contact training warrants attention because of higher overall injury and head impact risk, yet players must develop physical, technical, and mental skills to withstand the demands of the game. To help coaches manage contact loads in professional rugby, World Rugby and International Rugby Players convened an expert working group. They conducted a global survey with players to develop contact load guidelines. This commentary aims to describe the contact load guidelines and their implementation, and identify areas where future work is needed to support their evolution.

Rugby union became a professional sport in 1995, resulting in players and coaches having time and resources to train harder for longer. In professional rugby, the term ‘Load’ is commonly used to refer to the physical and non-physical demands placed on players, which encompass both rugby-related and non-rugby-related stressors and is defined as the sum of these stressors.^[1]^

In November 2014, World Rugby convened an ‘expert group’ to define the loads encountered by professional players and identify the possible implications for their physical and mental health. This group, comprising coaches, rugby administrators, player representatives, sports medicine and sports science practitioners, identified that, while match exposure is only 5–11% of total player exposure to rugby activity, the match injury rate is ~27 times higher than that of training.^[1]^

Given the greater exposure to training activity than match play and the relative level of control practitioners have over the duration and intensity of these activities, the working group recommended optimising training behaviours, specifically managing training load.

Since this meeting, several neuromuscular and tackle technique training programmes have been developed, with positive findings for their efficacy.^[2,3]^ Even with these programmes, injury prevention efforts in rugby still largely focus on interventions during matches. Strategies have included limits on match play in the professional game; for instance, the England Rugby Football Union imposes a limit of 30 full games or 35 match involvements (>20mins) per player per season in response to research showing that ≥31 match involvements are associated with a higher injury burden.^[4]^ Given the particular focus on concussion in the sport, several law variations to reduce tackle height in the community game and law applications to lower tackle height in the elite game have been implemented globally.^[5]^

In 2020, World Rugby and the International Rugby Players identified a need for more information and resources on load. It was felt that contact training needed special attention given the relatively high injury incidence associated with it compared to other training types, which must be balanced against the requirement to develop and maintain the physical, technical, and mental skills to perform during play.^[6]^ This was further supported by the fact that contact training poses the greatest risk for head impacts, and rugby’s concussion welfare efforts seek to reduce exposure to all unnecessary head impacts.

World Rugby and the International Rugby Players conducted a global survey of current professional game training practices to better understand how players engage in contact during training.^[7]^ Game, science, and medical expert review of these data led to the development of the contact load guidelines. These are intended to provide coaches and practitioners with guidance on planning, allocating and managing contact loads during the in-season in professional rugby.^[7]^ This commentary aims to describe the contact load guidelines and their implementation, and to identify areas where future work is needed to support their evolution.

## Contact load guidelines

The contact load guidelines outline four interrelated elements of contact training that must be managed. These aspects have been developed based on the FITT-acronym (Frequency, Intensity, Time and Type), which is commonly used for prescribing exercise and managing workload in sports.^[8]^ Each element is described below, along with methods that may be used to quantify and monitor player load (Table 1). These have typically been quantified subjectively and more recently, instrumented mouthguard technology has become available for use in rugby and offers potential as a proxy measure for the objective quantification of contact load.

## Practical implementation of guidelines

While all four contact elements are important to consider, contact *intensity* and *volume* are the easiest to measure. As such, it was recommended that *‘contact index’*, a variation of training load (RPE x duration), which is the product of *‘contact intensity’* (rated by the player on a 1–10 contact intensity scale) and *‘contact volume’* (minutes of contact exposure in a session), be documented as part of an ongoing load management strategy.

Contact load can broadly be subdivided into *full contact*, *controlled contact*, and *live set piece play*, where the key difference is contact intensity (Table 2). No optimal weekly structure of contact load has been identified; however, the expert working group has proposed several principles for each contact type.

**Fig. 1:**
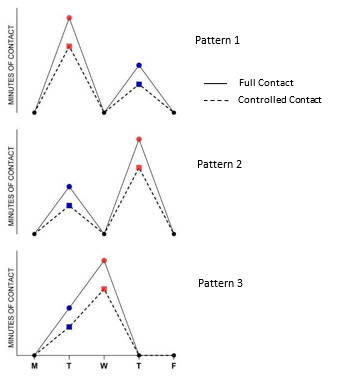
Three proposed weekly patterns of contact load during an in-season week, depicting no contact on a Monday or Friday and primary (red symbol) and secondary (blue symbol) contact load days.

Data from the survey identified three common patterns of contact exposure in the elite training week that respect these principles and enable flexibility to work around team and travel commitments (Figure 1).

For an in-season week in which the match is played on a Saturday, the first principle is that Mondays should have no *full-contact* and very low *controlled contact* and *live set piece* contact, to facilitate recovery from the previous match. Second, two of the remaining three days in the week should be selected as the primary and secondary load days where all contact training is performed. Third, Friday always represents a very low contact load day. The expert group advised that at most 55 minutes of total contact should be scheduled in a week, consisting of 15 min of *full contact* and 40 min of *controlled contact* per week (Table 2 and Figure 1). It was recommended that *controlled contact* and *full contact* are included in the same session, with *controlled contact* used as part of the progression to *full contact.*

When examining the distribution of reported time spent in contact training by the survey respondents, we observe that the recommended limits for *full contact* and *controlled contact* align closely with the reported median values (Table 2). Of the survey respondents, 66% of them engage in less than 24 minutes average of *full contact* training. If a limit of 15 minutes is implemented, it becomes evident that 50% of the respondents are already complying with this limit, implying that 50% would need to reduce their contact exposure to meet the recommendation. When we apply a similar approach to *controlled contact* and *live set piece* training, adhering to the recommended limits would require 50% of this group to decrease their *controlled contact* exposure, and 67% to decrease their participation in *live set pi*ece training. It is essential to exercise caution when interpreting the self-reported set piece training data since such training sessions can vary significantly in terms of contact intensity and, in the scrum specifically, the demands on different for playing positions (Table 2).

These guidelines should be applicable to most players, but because individual players respond differently to a given load based on personal characteristics, it is crucial to monitor each player’s response and adapt the load accordingly. There are also several contextual factors to consider that may require a player to be prescribed more or less contact from the outset. A player’s position, the previous week’s match exposure, playing experience and injury status are the primary considerations. Typically, older and more experienced players, players with higher recent match exposure, and those reporting minor injuries, should do less contact training in a week than their counterparts. Finally, it is important to remember that the total load experienced by a player is a combination of all physical and non-physical stressors and thus contact load is only one component of the stressors. As such, appropriate management of contact load is only possible when done as part of a broader load management strategy.

## Limitations and future work

The expert group created the guideline to help coaches and practitioners manage contact load during the in-season while providing players with necessary physical and performance preparations. There are several areas where future research is needed to progress the evolution of these guidelines.

Objective data are necessary to measure the four elements of contact load, and wearable devices like instrumented mouthguards could provide information on both the contact load and head acceleration exposure (HAE) of contact training activities. These mouthguards monitor head accelerations resulting from direct contact or movement of the head in response to body impacts. This data can identify activities with high contact load or HAE risk, as well as the unintended consequences of reduced exposure.^[9]^ It would also allow for the tracking of cumulative load, over a week and a season which when examined in relation to injury rates, would provide valuable information on optimal contact load for match readiness. Global Positioning Technology (GPS) tools have offered the prospect of documenting load through the analysis of their acceleration data. However, they lack the resolution necessary to accurately describe the forces experienced, and thus, their use for contact load monitoring, is limited.^[11,12]^ In contrast to GPS, which measures all movements, iMGs are only triggered through contact and while the mechanism of contact cannot be identified from the HAE alone and requires video review because the device is triggered by contact alone, it does offer the potential to track exposure to contact load. Given the suggested limits which are expected to lead to a decrease in the amount of time spent contact training for most participant players, we could expect this to be accompanied with a reduction in the occurrence of HAEs. To illustrate this, a hypothetical scenario is presented: if the initial rate of HAEs during full contact training was two per player per minute, then a player who previously engaged in 24 minutes of training (resulting in 48 HAEs) would now experience 30 HAEs in 15 minutes of training. This represents a reduction of 18 out of 48 HAEs or 36%. Precisely quantifying these reductions is just one of the many advantages or benefits of incorporating iMG data into training.

To support the collection of objective data, to date World Rugby has made iMGs available to all players across four professional male and four professional female competitions for one season, and two community level (u13 – adult) seasons. Although World Rugby is facilitating the technological resources for data capture, it is important to acknowledge that there may be an associated time commitment for staff to handle and act on the data. This is particularly relevant since the technology and the related data may be unfamiliar to many staff members. Consequently, it is advisable to conduct further research to comprehend this time burden and pinpoint strategies to reduce it. Even with objective data available, training prescriptions will always contain an element of subjectivity; thus to develop safe and effective training practices, the relationship between coach and player perceptions of training and objective measurements must be established. The evolution of contact load guidelines should involve players, game officials, and medical stakeholders to ensure practicality and adherence.

The global survey revealed a large range in the time players felt they spent doing contact training in a week (0 – 540 minutes of total contact training in a week; 0 – 145 minutes of this time comprising full contact training). It is possible that recall bias impacted the accuracy of the times reported. However, this wide range reveals a variety of training practices and significant divergence in how players interpret contact categories. To facilitate accurate monitoring of contact training exposure and to enable data across studies to be comparable, there is a need for standardised definitions of contact training to be developed.

The contact load guidelines treat set piece training as additional to other contact loads and guide the implementation of *live set piece* contact specifically. Due to the variability of contact intensity within a set piece training session and between playing positions, further research is required to determine how much set piece training, at various intensities, currently occurs.

It is worth highlighting that as *set piece* training is almost exclusively for forwards, they will experience more total weekly contacts than backs. A limitation of the survey was that it did not identify the position of the respondents. Backs typically cover greater distances at higher speeds during match play, while forwards are involved in significantly more contact events.^[13]^ Therefore it is likely that players will receive position-specific training, and as a result, future data collected to support contact training recommendations must take a player’s position into account.

Although the fundamental principles of contact training are the same for men and women, with only 11% of survey responses from female players, further research is required to establish the optimal contact load for women. Even at the elite level, female players often have a different depth of experience than their male counterparts, which has implications for the balance sought between optimal training contact load for match preparation and injury risk. It is thus important that efforts to reduce overall contact load in the women’s game account for the potential lower training age and are balanced with sufficient training exposure to improve players’ technical ability. Particular attention should also be paid to the potential impact of the menstrual cycle phase on injury risk during contact. Additional research is necessary to determine the optimal exposure and patterns of implementation of contact training in other levels and age grades.

Finally, the current guidelines pertain specifically to the in-season period and do not cover the preseason period, where the requirements for contact load are likely to differ. As is required for all training domains, contact load should be delivered and managed in a progressive and periodised fashion, with the preseason focus different from that of the in-season focus. Further research is, however, required to develop contact load guidelines for this period of the season.

## Conclusion

Preventing injuries and optimising performance requires effective management of contact load. To improve the guidance given to coaches on managing contact load, further research is needed to refine and enhance the guidelines. Primarily, acquiring objective data is crucial to understanding the risks associated with contact training, and stakeholder engagement is necessary to ensure that any resulting interventions are practical and feasible to implement.

## Figures and Tables

**Table 1:** The four elements of contact training and subjective and objective methods for assessing these elements in a training session/drill

Contact element	Description	Subjective measurement	Objective measurement
**Intensity**	The magnitude of contact events within a session/drill. Primarily a function of speed into contact, size of the area of drill, and the player’s application of force.	By rating the magnitude of impacts in a session on a 1 to 10 contact intensity scale: 1 = almost zero contact; 10 = match equivalent contact intensity.	Quantified using data obtained from instrumented mouthguards, or GPS technology. Size of the area of the drill.
**Density**	Frequency of contacts within a session, drill, or unit of time.	Graded as ‘high’, ‘medium’ or ‘low’: *High* = high frequency, or short time between contacts; *Low* =low frequency, or longer periods of time between contacts. *[Short vs. long periods of time is subjective and at the discretion of the coach]*	Quantifying the number of contacts per minute through video analysis, mouthguard or GPS technology.
**Unpredictability**	Degree to which a player can anticipate their direct opponent’s actions during contact activities; a function of how controlled or structured a drill and session.	Graded as ‘high’, ‘medium’ or ‘low’: *High* = players must react to opponent’s unknown actions; *Medium* = some control is imposed by coaches; *Low* = high degree of structure in session	No reliable objective metric. Best assessed subjectively as pertains to the level of structure or planning in session.
**Volume**	Total amount of contact within a session/drill	Always measured objectively, either indirectly or directly	*Indirectly*: minutes of contact exposure time. *Directly*: quantifying the number of contacts using data obtained from instrumented mouthguards.

**Table 2:** Weekly in-season contact load recommendations

	Full contact	Controlled contact	Live set piece contact
**Description**	Unrestricted, body on body, without the use of shields/pads	Restricted in terms of speed and force, incorporating shields/pads	Scrums, lineout, kick-off receiving and mauls, fully contested at near match intensity
**Subjective measurement**	Contact intensity scale 8 to 10	Contact intensity scale 7 or less	Contact intensity scale 8 to 10
**Maximum total weekly volume**	15 minutes	40 minutes	30 minutes
**Average (range) weekly volume of survey respondents**	24 (0 – 150) minutes	55 (0 – 240) minutes	50 (0 – 280) minutes
**Median (IQR) weekly volume of survey respondents**	15 (5 – 30) minutes	45 (25 – 78) minutes	40 (5 – 75) minutes

## Data Availability

The authors confirm that the data supporting the findings of this commentary are available within the article.
